# Whole-Genome Sequencing and Phenotypic Drug Susceptibility Testing of Bedaquilin, Delamanid, Pretomanid, and Linezolid in Drug-Resistant *Mycobacterium tuberculosis* from a Single Institute in South Korea

**DOI:** 10.3390/pathogens15030320

**Published:** 2026-03-16

**Authors:** Hyun-Woo Choi, Yoo-Ree Kang, Eun-Soon Son, Kyungsik Choi, Myungsun Cho, Young Jin Kim, Seo A Lee, Jin Young Lee, Jee Hey Kim, Seon Joo Kang, Seung-Jung Kee, Jong Seok Lee, Hee Joo Lee

**Affiliations:** 1 Department of Laboratory Medicine, Chonnam National University Medical School and Hospital, Gwangju 61496, Republic of Korea; wiseltree@gmail.com (H.-W.C.); sjkee1968@naver.com (S.-J.K.); 2 Laboratory Medicine Center, SD Medical Research Institute, Yongin 17013, Republic of Korea; yuree7217@naver.com (Y.-R.K.); leejy@sdmri.co.kr (J.Y.L.); sophia7322@sdmri.co.kr (J.H.K.); drk428@sdmri.co.kr (S.J.K.); 3 Microbiology Research Section, International Tuberculosis Research Center, Changwon 51755, Republic of Korea; fin2929@naver.com (E.-S.S.); cosmosljs@gmail.com (J.S.L.); 4 Laboratory Medicine Center, Korea National Tuberculosis Association, Seoul 06763, Republic of Korea; cando1968@hanmail.net (K.C.); lj0205@nate.com (M.C.); 5 Department of Laboratory Medicine, Kyung Hee University College of Medicine, Kyung Hee University Hospital, Seoul 02447, Republic of Korea; khmclab@gmail.com; 6 Department of Biomedical Sciences, Graduate School of Chonnam National University, Gwangju 61186, Republic of Korea; m00n5518@daum.net

**Keywords:** *Mycobacterium tuberculosis*, drug-resistant tuberculosis, bedaquiline, delamanid, pretomanid, linezolid

## Abstract

Multidrug-resistant tuberculosis is a major global health concern. Newer agents, including bedaquiline (BDQ), delamanid (DLM), pretomanid (PMD), and linezolid (LZD), are essential for treatment; however, the resistance mechanisms of these drugs remain poorly understood in South Korea. This study aimed to investigate correlations between phenotypic and genotypic resistance to these drugs using 49 clinical *Mycobacterium tuberculosis* isolates collected in South Korea between 2017 and 2022. The minimum inhibitory concentrations were determined using the 7H9 broth microdilution method, and whole-genome sequencing (WGS) results were compared with the May 2024 World Health Organization (WHO) mutation catalogue. Phenotypic drug susceptibility testing (pDST) revealed elevated MICs to BDQ in 12 isolates (24.5%), DLM in nine (18.4%), and PMD and LZD in two each (4.1%). No Group 1 or 2 resistance-associated mutations were detected in BDQ-, PMD-, or LZD-elevated-MIC isolates. A Group 2 mutation (*fbiC*_LoF) was observed in one DLM-elevated-MIC isolate, whereas *fbiC*_p.Ala855fs (WHO Group 2) mutations occurred in four susceptible isolates. These findings suggest resistance mechanisms beyond the current WHO catalog. Discrepancies between pDST and WGS highlight the need for integrated diagnostics and reinforce the importance of ongoing surveillance and refinement of mutation classification systems to improve genotypic resistance prediction.

## 1. Introduction

Tuberculosis (TB) is an infectious disease caused by *Mycobacterium tuberculosis.* It remains a major global health challenge, contributing substantially to morbidity and mortality. According to the World Health Organization (WHO) Global Tuberculosis Report 2025, TB remains one of the leading cause of death from a single infectious agent worldwide [1]. In South Korea, the total number of reported TB cases in 2023 was 19,540 (38.2 cases per 100,000 population), reflecting a 4.1% decrease from 20,383 cases (39.8 per 100,000) in 2022 [2]. Although the incidence of TB is decreasing, drug-resistant TB remains a critical challenge.

The emergence of multidrug-resistant TB (MDR-TB) and extensively drug-resistant TB (XDR-TB) complicates treatment, highlighting the urgent need for new therapeutic strategies. MDR-TB is defined as TB resistant to at least rifampicin (RIF) and isoniazid (INH), which are two key first-line TB drugs. Some cases of MDR-TB may develop additional resistance to fluoroquinolones, classifying them as pre-extensively drug-resistant TB [1]. XDR-TB is a more severe form of resistance characterized by resistance to RIF (and potentially INH), at least one fluoroquinolone (levofloxacin [LVX] or moxifloxacin [MFX]), and at least one additional Group A drug (such as bedaquiline [BDQ] or linezolid [LZD]). In 2023, 175,923 patients worldwide were diagnosed with MDR-TB or rifampicin-resistant TB (RR-TB). In South Korea, 551 people were diagnosed with MDR/RR-TB, accounting for 2.8% of all TB cases [2]. The persistent burden of MDR-TB and XDR-TB raises major public health concerns as these forms of TB not only prolong disease transmission but also lead to higher treatment costs and increased mortality rates. Therefore, the development of new drugs, optimized treatment regimens, and robust global surveillance are essential to effectively combat drug-resistant TB and prevent its further spread.

Key agents in the treatment of MDR-TB, particularly in short-course regimens, such as BPaL(M) and MDR-END, include BDQ, delamanid (DLM), pretomanid (PMD), and LZD. These drugs have distinct mechanisms of action: BDQ targets ATP synthase and disrupts energy production [3]; DLM and PMD interfere with mycolic acid biosynthesis [4]; and LZD inhibits protein synthesis by binding to the 50S ribosomal subunit [5]. The BpaL(M) regimen consists of BDQ, PMD, and LZD with the optional addition of MFX. In contrast, the MDR-END regimen comprises LVX, DLM, LZD, and pyrazinamide. Analysis of resistance to BDQ, DLM, PMD, and LZD used in MDR-TB treatment is crucial, as they significantly influence treatment and outcomes. However, research on BDQ-, DLM-, PMD-, and LZD-related drug resistance mutations is lacking compared to that on traditional anti-TB drugs, such as RIF and INH. Furthermore, research on drug resistance mutations associated with DLM and PMD remains limited, with only a small number of studies having investigated their molecular mechanisms [6].

Therefore, this study aimed to comprehensively identify and compare BDQ-, DLM-, PMD-, and LZD-resistance-related mutations in 49 clinical isolates of *M. tuberculosis* using phenotypic drug susceptibility testing (pDST) and genotypic drug susceptibility testing (gDST). Additionally, we sought to compare gDST results with drug resistance associations based on the 2024 updated version of the World Health Organization (WHO) *M. tuberculosis* mutation catalogue.

## 2. Materials and Methods

### 2.1. Specimen Collection

*Mycobacterium tuberculosis* isolates cultured from sputum samples of patients with active pulmonary TB were obtained from the Chonnam National University Hospital, Gwangju, South Korea, between January 2017 and December 2022. Among the 141 isolates, those that failed to grow or showed contamination during subculture were excluded. From the remaining isolates, 49 drug-resistant isolates were randomly selected and included for analysis. This observational cohort study was reviewed and approved by the Institutional Review Board of Chonnam National University Hospital (CNUH IRB) (IRB approval number: CNUH-2025-156). As the study involved retrospective analysis of anonymized *M. tuberculosis* isolates obtained from sputum samples, the requirement for informed consent was waived by the IRB. The study was conducted in accordance with the principles of the Declaration of Helsinki.

Drug-resistant *M. tuberculosis* clinical isolates were sequentially cultured in Middlebrook 7H9 broth (BD Biosciences, Franklin Lakes, NJ, USA) and 3% Ogawa II Medium (Asanpharm, Seoul, Republic of Korea) before use. Bacterial colonies in the logarithmic growth phase were collected and used for minimum inhibitory concentration (MIC) determination and whole-genome sequencing (WGS) analysis.

### 2.2. pDST: MIC Determination of BDQ, DLM, PMD, and LZD Using the Resazurin Microdilution Assay

The MIC for each drug was determined using a resazurin-based broth microdilution method in Middlebrook 7H9 broth. All isolates, including M. tuberculosis H37Rv (ATCC 27294), were first grown to mid-log phase (OD600 = 0.4–0.6). Cultures were subsequently diluted to achieve the required inoculum for MIC testing, corresponding to a calculated OD600 range of 0.0008–0.0064. These values represent dilution-based estimates derived from mid-log-phase cultures rather than direct spectrophotometric measurements below the linear detection range. The final inoculum was adjusted to approximately 10^5^ CFU per well, consistent with standard broth microdilution protocols.

Resazurin was added after the incubation period as a post-incubation viability readout, rather than during the incubation phase. The plates were incubated at 37 °C for 7 days, after which resazurin was added and MICs were determined according to our predefined experimental protocol. Wells without resazurin color conversion at this time point were interpreted as showing no detectable growth under the tested conditions. A color change from blue (oxidized form) to pink (reduced form) indicated bacterial growth, whereas no color change indicated bacterial growth inhibition. The resazurin colorimetric assay was used as the primary viability endpoint, and confirmatory colony-forming unit (CFU) enumeration was not performed. Growth was assessed by comparing the colorimetric results with those of the positive control (drug-free medium) and negative control (medium without bacterial inoculation) to determine the MIC values.

All MIC experiments were performed in technical dupluicate. In pDST, the standard quality-control strain *M. tuberculosis* H37Rv was tested under the same experimental conditions as the clinical isolates and served as an internal wild-type control. The measured MICs for H37Rv were as follows: BDQ 0.125 mg/L, DLM 0.06 mg/L, PMD 0.25 mg/L, and LZD 0.6 mg/L. Elevated MICs were defined according to published epidemiological cutoff values exceeding the wild-type MIC distribution: BDQ ≥ 0.5 mg/L [7], DLM ≥ 0.125 mg/L [8], PMD ≥ 4 mg/L [9], and LZD ≥ 1.2 mg/L [10].

### 2.3. gDST: WGS Method

DNA was extracted from 49 fully cultured *M. tuberculosis* isolates grown in broth media at 37 °C. Genomic DNA was extracted using the SPINeasy DNA Kit for Tissue and Bacteria with Lysing Matrix (MP Biomedicals, Irvine, CA, USA; Cat No. 116530050) according to the manufacturer’s protocol. The extracted DNA was quantified using the Qubit dsDNA HS assay kit (Thermo Fisher Scientific, Waltham, MA, USA), and purity was assessed by measuring the A260/A280 ratio using a Nanodrop spectrophotometer (Thermo Fisher Scientific). The integrity of genomic DNA was further confirmed using agarose gel electrophoresis. Sequencing libraries were prepared using a Nextera XT DNA Library Preparation Kit (Illumina, San Diego, CA, USA). One nanogram of genomic DNA was subjected to tagmentation, in which DNA fragmentation and adapter ligation occurred in a single step. Fragmented DNA was purified using AMPure XP magnetic beads (Beckman Coulter Life Sciences, Indianapolis, IN, USA) to remove short fragments, followed by limited-cycle PCR amplification and normalization. The final purified libraries were quantified by qPCR using the KAPA Library Quantification Kit (KAPA Biosystems, Wilmington, MA, USA), and their quality was assessed using the TapeStation D5000 ScreenTape system (Agilent Technologies, Santa Clara, CA, USA). WGS was performed using the Illumina MiSeq platform (2 × 250 bp) and Ion Torrent S5 XL system (Thermo Fisher Scientific, Waltham, MA, USA). The use of two sequencing platforms was based on institutional availability and sequencing timelines during the study period. To ensure consistency, all sequencing data generated from both platforms were processed using the same bioinformatics pipeline, variant calling criteria, and quality-control thresholds. No systematic differences in the detection of resistance-associated mutations were observed between the Illumina MiSeq and Ion Torrent S5 XL platforms. All analyses, including variant calling and genome assembly, were performed using short-read data.

### 2.4. Sequencing Data Processing

WGS analysis was performed using the CLC Genomics Workbench (version 23.0.4, Qiagen, Hilden, Germany) in accordance with the 2024 updated version of the WHO *M. tuberculosis* mutation catalogue and its association with drug resistance. Raw sequencing reads were generated in the FASTQ format and subjected to quality-control assessment using FastQC. Low-quality bases and adapter sequences were trimmed using Trimmomatic software (version 0.39). Reference-based assembly was conducted using the *M. tuberculosis* H37Rv genome (NC_000962.3) as a reference, with a length fraction of 0.5 and a similarity fraction of 0.8. The breadth of genome coverage against the *M. tuberculosis* H37Rv reference genome ranged from 96% to 100%, with an average of approximately 98% across all isolates, ensuring sufficient coverage for accurate variant detection. Variant calling was performed using the Fixed Ploidy Variant Detection tool in the CLC Genomics Workbench, with a minimum read depth of 10× and a variant-allele frequency threshold of 0.9. The tool outputs both homozygous and heterozygous calls based on variant-allele fraction and genotype proportions. Zygosity was retained during analysis to allow detection of hetero-resistance, and several variants were observed, with intermediate allele frequencies consistent with mixed-strain populations. The identified variants were annotated using the WHO catalog to determine their associations with drug resistance.

## 3. Results

### 3.1. Drug Resistance Classification and Lineage Distribution of Isolates

**Figure 1:**
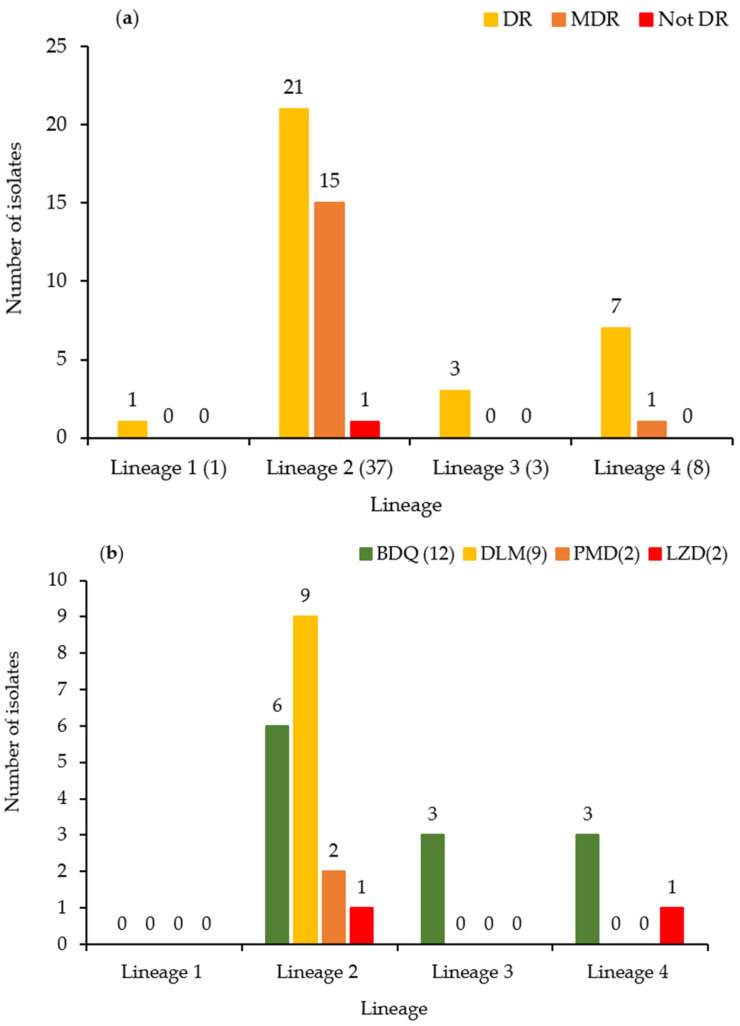
DR characteristics according to lineage and lineage distribution of *Mycobacterium tuberculosis* isolates exhibiting elevated MICs to BDQ, DLM, PMD, or LZD. (**a**) The bar chart represents the distribution of DR characteristics across different lineages. Lineage 2 has the highest occurrence in DR and MDR cases, whereas Lineages 1 and 3 exhibited lower counts. Absolute numbers of the isolates are displayed above each bar. (**b**) Bars represent the number of isolates exhibiting elevated MICs to BDQ, DLM, PMD, and LZD within Lineages 1–4. The number above each bar indicates the absolute number of isolates with elevated MICs to the corresponding drug within each lineage. The numbers next to each lineage (n) indicate the number of isolates out of the 49 isolates classified into that lineage, whereas the numbers next to each drug (n) indicate the number of isolates with elevated MICs to the respective drug. BDQ, bedaquiline; DLM, delamanid; PMD, pretomanid; LZD, linezolid; DR, drug-resistant; MDR, multidrug-resistant.

Among the 49 isolates, 32 (65.3%) were classified as drug-resistant, 16 (32.7%) as MDR, and one as not drug-resistant (2.0%). The isolates were assigned to four major lineages: Lineage 1 (Indo-Oceanic family), Lineage 2 (East Asian/Beijing family), Lineage 3 (East African–Indian family), and Lineage 4 (Euro-American family). Lineage 2 (Beijing family) accounted for the most isolates (37 isolates; 75.5%), followed by Lineage 4 (8 isolates; 16.3%), Lineage 3 (3 isolates; 6.1%), and Lineage 1 (1 isolate; 2%) (Figure 1a,b).

### 3.2. pDST Results

**Figure 2:**
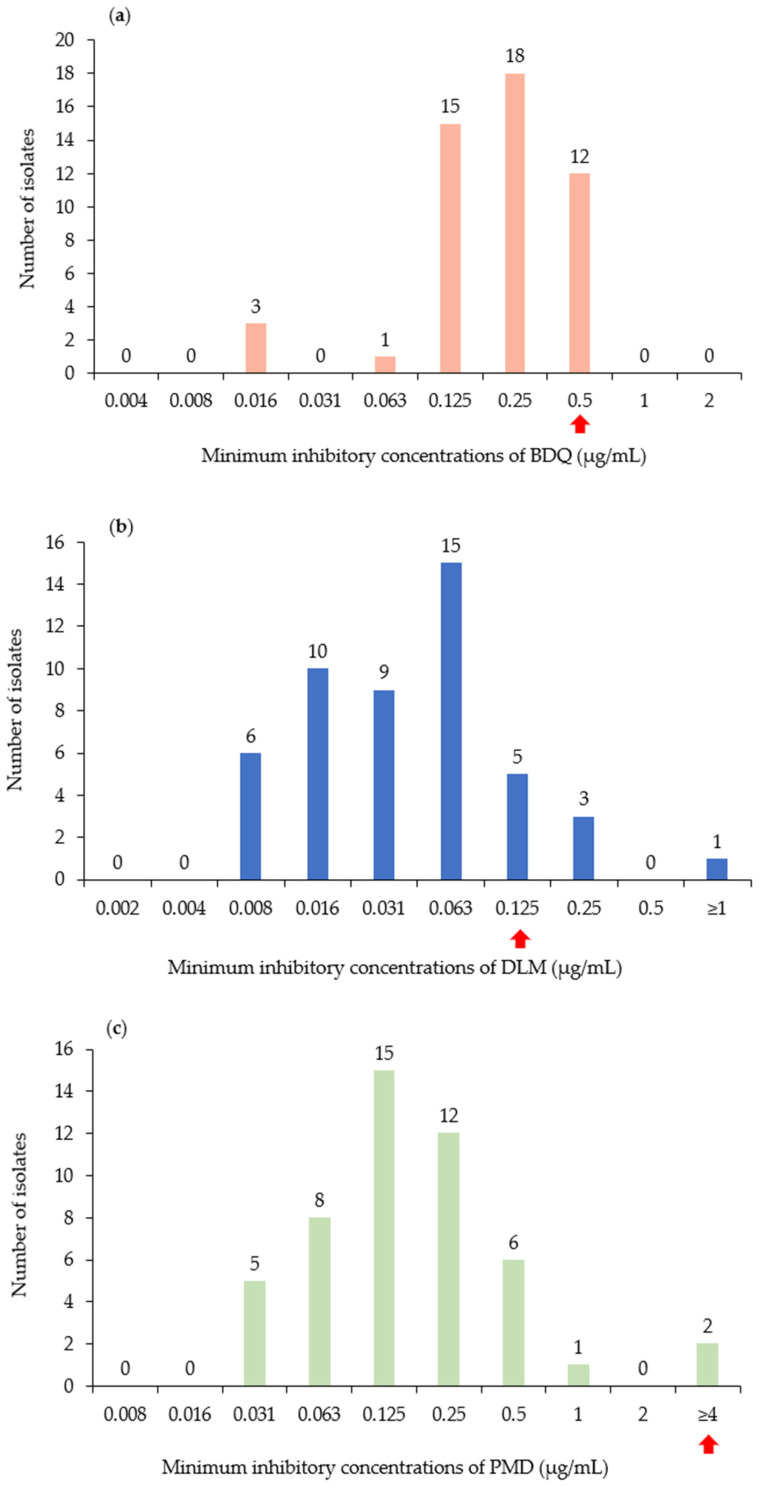

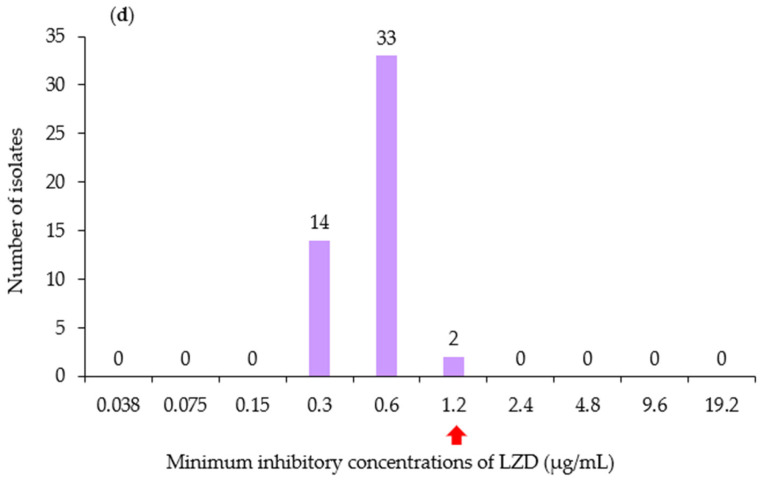
pDST results in 49 *Mycobacterium tuberculosis* isolates with elevated MICs to BDQ, DLM, PMD, and LZD. The MICs of the cultured isolates were determined using the microplate dilution method for each tested drug. The resazurin microdilution assay was performed using serial twofold dilutions across 1 to 10 concentration levels, and the full range of dilution steps used for MIC determination is presented in the figure. The concentration ranges for each drug were as follows: (**a**) BDQ (0.004–2 µg/mL); (**b**) DLM (0.002–1 µg/mL); (**c**) PMD (0.008–4 µg/mL); and (**d**) LZD (0.038–19.2 µg/mL). Resistance was determined when cultured at drug concentrations of BDQ ≥ 0.5 µg/mL, DLM ≥ 0.125 µg/mL, PMD ≥ 4 µg/mL, and LZD ≥ 1.2 µg/mL. Arrows indicate the MIC threshold used to define elevated MICs for each drug. pDST, phenotypic drug susceptibility testing; BDQ, bedaquiline; DLM, delamanid; PMD, pretomanid; LZD, linezolid; MIC, miminum inhibitory concentration.

According to the pDST, 12 isolates (24.5%) exhibited elevated MICs to BDQ (Figure 2a), nine isolates (18.4%) to DLM (Figure 2b), and two isolates (4.1%) each to LZD and PMD (Figure 2c,d). Analysis of lineage distribution among isolates exhibiting elevated MICs revealed that, among the 12 isolates exhibiting elevated MICs to BDQ, six isolates belonged to Lineage 2, whereas three isolates each belonged to Lineage 3 and Lineage 4. Notably, only three of the 49 isolates were classified as Lineage 3, with all three isolates found in the BDQ-elevated-MIC group. All nine isolates elevated MICs to DLM and two isolates with elevated MICs to PMD were classified as Lineage 2. Among the two isolates with elevated MICs to LZD, one belonged to Lineage 2 and the other to Lineage 4 (Figure 1b).

### 3.3. gDST: WGS Results

WGS analysis was performed on all isolates, and the results were compared with the 2024 updated version of the WHO *M. tuberculosis* mutation catalogue. No mutations classified under WHO Group 1 (associated with resistance) or Group 2 (associated with resistance—interim) were detected in LZD-, BDQ-, or PMD-elevated-MIC isolates. However, for DLM, one of the nine elevated-MIC isolates (Sample No. 64) harbored the *fbiC*_LoF mutation, which is classified under WHO Group 2. Additionally, four of the 40 susceptible isolates (Sample Nos. 20, 21, 43, and 67) carried the *fbiC*_p.Ala855fs mutation, also classified as WHO Group 2 (Table 1). To provide additional context for these discrepancies, mutations detected in BDQ-, DLM-, and LZD-associated genes were further summarized according to the WHO confidence-grading system beyond Groups 1 and 2, including Group 3 (uncertain significance), Group 4 (not associated with resistance—interim), and Group 5 (not associated with resistance) (Table 2). Under this expanded classification, a greater number of mutant genes were identified. Notably, the BDQ-associated mutations *mtrB*_Met517Leu (Group 3), *mmpL5*_Ile948Val (Group 4), and *Rv1979c*_c.-129A > G (Group 4) were found in all 49 isolates (Table 2).

Additional mutations not listed in the WHO mutation catalog were identified in some resistant isolates. Mutations in *glpK* were identified in four of the 12 BDQ-elevated-MIC isolates. Mutations in *fbiC* were detected in one isolate each among the nine DLM-elevated-MIC and two PMD-elevated-MIC isolates. No LZD-resistance-associated mutations were detected (Table 3). Among the 27 drug-susceptible isolates, *Rv1979c* and *glpK*, which may be associated with BDQ resistance, were identified in six and three isolates, respectively. The *fbiC* gene mutation, which is associated with DLM and PMD resistance, was detected in six isolates. Additionally, mutations were identified in three LZD-resistance-related genes: *rrl* (two isolates), *rplD* (one isolate), and *rplV* (one isolate) (Table 4).

## 4. Discussion

In this study, the phenotypic and genotypic resistance to BDQ, DLM, PMD, and LZD were investigated in 49 *M. tuberculosis* clinical isolates collected in South Korea between 2017 and 2022. Most isolates belonged to Lineage 2 (Beijing family), followed by Lineage 4. This distribution is consistent with those reported by previous studies conducted in South Korea, where approximately 70–80% of isolates belong to Lineage 2 and 20–30% to Lineage 4 [11,12]. Lineage 2 has been reported to be epidemiologically and clinically important, given its association with increased transmissibility, sustained circulation, and a propensity for drug resistance in multiple settings [13]. Previous studies have further indicated that Lineage 2 plays a key role in the propagation of MDR-TB, particularly in East Asia, including South Korea [14,15]. Therefore, the predominance of Lineage 2 among isolates exhibiting elevated MICs in this study may be interpreted as underscoring the importance of continued phenotypic and genomic surveillance within this lineage, particularly given the expanding clinical use of BDQ, DLM, and LZD.

Our cohort comprised drug-resistant clinical isolates collected from a single institute rather than a population-based sample of unselected strains. This enrichment for resistance phenotypes may have shifted the observed MIC distribution upward compared with studies reporting wild-type distributions from largely drug-susceptible collections. Phenotypic MIC elevation was detected in 12 isolates for BDQ, nine for DLM, and two each for PMD and LZD. However, most isolates with elevated MICs did not harbor resistance-associated mutations currently listed in the 2024 WHO mutation catalog. This discordance between pDST and WGS underscores the complexity of resistance mechanisms and suggests that the existing mutation catalog may not fully account for all clinically relevant resistance phenotypes. These observations support the need to further refine phenotypic thresholds and genotypic classification systems and emphasize the importance of continued genomic surveillance to identify additional or as-yet unrecognized mechanisms of resistance.

The resistance rates observed in this study were higher than those previously reported, particularly for BDQ and DLM [9,16]. These differences may be attributable to variations in anti-TB drug usage histories among patients, as well as the timing of isolate collection during the period when these drugs were being newly introduced or increasingly adopted in MDR-TB treatment protocols [4,17]. Alternative mechanisms, such as mutations in uncataloged loci (e.g., *glpK* and *fbiD*) [18,19], efflux pump regulation [20], and epigenetic or transcriptional changes not captured by short-read sequencing [21,22,23], could also explain these findings. Moreover, the possibility that current MIC thresholds for novel drugs may not fully capture borderline resistance should be considered [16]. This study highlights the importance of region-specific monitoring of resistance trends, particularly in countries where new drugs are being integrated into national TB programs.

The H37Rv reference strain consistently demonstrated a BDQ MIC of 0.125 mg/L, which falls within the reported wild-type distribution range (0.03–0.125 mg/L) described in previous REMA/MABA studies [24,25]. Although slightly higher than the modal value reported in some studies (0.03–0.06 mg/L) [26,27], this value remains within the accepted wild-type range. Importantly, isolates with MIC values within one dilution of the wild-type range (e.g., 0.05–0.06 mg/L) were not classified as resistant but were considered within the expected variability of the microdilution assay. Elevated MICs were defined only for isolates with MIC values ≥0.5 mg/L, exceeding both the wild-type distribution and WHO-reported epidemiological cutoff values [8]. Based on these criteria, 12 isolates were classified as having elevated BDQ MICs by pDST. Despite the elevated BDQ MICs, WGS did not detect mutations in WHO Group 1 or 2 resistance-associated loci. *Rv0678*, *pepQ*, and *Rv1979c* have been implicated in BDQ resistance [28,29,30,31]; however, many of these variants are still missing from the current WHO mutation catalog. This aligns with the current findings and those of other studies in which phenotypic resistance was not associated with known cataloged mutations, suggesting the involvement of unrecognized genetic determinants or regulatory mechanisms.

In contrast, WGS detected a Group 2 resistance mutation, *fbiC*_LoF, in one of the nine isolates with elevated MICs to DLM (Sample No. 64), while a frameshift mutation, p. Ala855fs, in *fbiC* was also present in four DLM-susceptible isolates (Sample Nos. 20, 21, 43, and 67). This discrepancy suggests that not all mutations confer phenotypic resistance and highlights the complexity of DLM resistance mechanisms [32,33,34]. Notably, many reported mutations associated with DLM resistance remain uncataloged by the WHO [35], suggesting the need to expand and refine current mutation classification systems. Methodological differences between this study and the WHO mutation catalog may also influence this observation. Some mutations identified in this study but absent from the WHO catalog may represent early or borderline resistance rather than true omissions.

pDST revealed that two isolates exhibited resistance to PMD; however, no recognized mutations were observed in the WGS analysis. Prior studies have identified potential resistance mutations in ddn (e.g., p.Tyr29del and p.Arg31fs); however, their classification remains provisional [36]. Other candidate mutations in *fgd1*, *fbiA*, *fbiB*, *fbiC*, and *fbiD* (*Rv2983*) have also been reported [9,18,37,38]. However, as PMD has only recently been introduced into clinical use, the related resistance mutations remain poorly characterized. Functional validation studies, such as allelic exchange or in vitro drug exposure experiments, are warranted to determine whether novel variants are involved in resistance.

In the pDST results of this study, among the nine isolates exhibiting elevated MICs to DLM, only one isolate simultaneously showed elevated MICs to PMD. Both DLM and PMD are nitroimidazole-class antituberculosis drugs that share a similar mechanism of action, as they require activation through the F420-dependent nitroreductase pathway (including *fbiA*, *fbiB*, *fbiC*, and *fgd1*) in *M. tuberculosis* [4]. Therefore, the possibility of cross-resistance between these two agents cannot be excluded from a mechanistic perspective.

Many studies have shown that cross-resistance between the two agents is not consistently detected, and that complete cross-resistance is not commonly observed [18,39,40]. This discrepancy may be attributed to the complex activation process of nitroimidazole-class drugs [41], in which the effects of individual agents can vary depending on the specific combination of genetic mutations and their expression levels [42,43]. Especially for MIC values near clinical cutoffs, interpretive uncertainty has been recognized [44], with test results potentially varying depending on lineage, assay method, and interpretive criteria, indicating that borderline MIC elevations may be context-dependent. Taken together, these results suggest that the discordant MIC patterns observed between DLM and PMD in this study are consistent with findings reported in the existing literature.

Previous studies on LZD resistance have reported that LZD-resistant isolates were not identified in cohorts from Indonesia [31] and Latvia [45]. In South Korea, LZD resistance has not been frequently observed [46], although sporadic cases have been reported worldwide [47]. Mutations in *rplC* and *rrl* have been associated with LZD resistance [26,48,49]. In this study, the two isolates exhibiting elevated MICs to LZD are more likely to reflect borderline values or assay-related variability rather than established resistance. Consistent with this interpretation, no WHO Group 1 or Group 2 resistance-associated mutations were identified. Although *rrl* variants were detected, these were also observed in isolates classified as susceptible. Importantly, the detected *rrl* variants were located outside the established LZD resistance hotspot in the 23S rRNA domain V [50], and were therefore interpreted as non-resistance-associated polymorphisms. The presence of these variants in both isolates with elevated MICs and those classified as susceptible further supports their interpretation as background polymorphisms rather than definitive resistance determinants. Following the 2023 World Health Organization guidelines and the 2024 revision of the Korean Tuberculosis Treatment Guidelines (5th edition) [51], the clinical use of BDQ and LZD as core drugs for the treatment of MDR-TB has expanded substantially. Given the expanding clinical use of BDQ and LZD, continued genomic surveillance is essential to monitor the emergence of resistance-associated mutations, detect early or borderline resistance patterns, and support the timely optimization of treatment strategies.

The discrepancies between the pDST and WGS results, particularly the absence of known resistance mutations in phenotypically resistant isolates, suggest potential limitations of both diagnostic methods. Although MIC-based pDST provides quantitative data, interpretation thresholds for novel drugs may still require refinement. Additionally, WGS using short-read sequencing may miss resistance mechanisms related to gene expression or epigenetic regulation. Future studies integrating transcriptomics, long-read sequencing, and functional genomics are essential to bridge these gaps.

This study has a few limitations. First, the sample size was relatively small, and all isolates were collected from a single region within South Korea, limiting the generalizability and ability to assess geographic or temporal variation. Second, in pDST, the absence of CFU-based validation and the use of a single incubation time point for MIC determination may not fully capture delayed growth in slow-growing isolates. Third, WGS was performed using short-read sequence data with a mininum read depth threshold of 10x. Although this coverage was sufficient for identifying dominent resistance associated mutations, it may have limited the detection of low-frequency varients or minor resistant subpopulations. Fourth, known WHO-cataloged resistance mutations were not observed in any of the BDQ-, PMD-, or LZD-elevated-MIC isolates, which may reflect limitations of WGS-based detection of structural variants or gene regulation. Finally, additional molecular analyses, such as RNA-seq, that may have further clarified the resistance mechanisms were not performed.

Despite these limitations, this study makes three key contributions. First, it provides new data on susceptibility patterns for four core anti-TB drugs (BDQ, DLM, PMD, and LZD) using both pDST and WGS, contributing to the limited body of research specific to the Korean context. Second, the absence of known resistance mutations in phenotypically elevated-MIC isolates supports the potential involvement of novel or unclassified mechanisms. Third, the discrepancy between the phenotypic and genotypic results highlights the need for updated pDST thresholds and the complementary use of WGS in clinical diagnostics. These findings emphasize the importance of region-specific surveillance and collaborative global efforts to improve resistance detection in MDR-TB management.

## 5. Conclusions

In conclusion, this study identified discrepancies between pDST and WGS results in *M. tuberculosis* clinical isolates with elevated MICs to BDQ, DLM, PMD, and LZD. These may be attributed to variations in sample characteristics, differences in prior drug exposure, limitations of current WGS-based detection methods, and the possibility of the existence of novel resistance-conferring mutations in unrecognized genes. Particularly, the lack of known mutations in the 2024 WHO mutation catalog in several phenotypically elevated MIC isolates highlights the need to expand our understanding of resistance mechanisms. Although the updated catalog includes a broader range of variants than previous versions, further global data integration and regular updates are essential to enhance the predictive power of genotypic diagnostics. To improve the accuracy of resistance detection, future research should identify uncatalogued and novel mutations using integrated approaches, such as long-read sequencing and transcriptomic analysis. Additionally, the critical concentration thresholds used in pDST should be reexamined using larger and more diverse datasets. These efforts are essential for refining drug resistance surveillance and optimizing treatment strategies for MDR-TB globally.

## Figures and Tables

**Table 1:** pDST and gDST results for LZD-, DLM-, BDQ-, and PMD-elevated-MIC isolates.

Sample Number	Treatment Status	pDST (MIC)				gDST
		BDQ (µg/mL)	DLM (µg/mL)	PMD (µg/mL)	LZD (µg/mL)	Mutations Classified Under Group 1 * and Group 2 ^†^ in the 2024 WHO Catalogue of Mutations in *Mycobacterium tuberculosis*
59	During treatment	0.5 (R)	0.125 (R)	0.25 (S)	0.6 (S)	-
139	Before treatment	0.5 (R)	0.25 (R)	0.5 (S)	1.2 (R)	-
17	During treatment	0.5 (R)	0.008 (S)	0.125 (S)	0.3 (S)	-
18	During treatment	0.5 (R)	0.031 (S)	0.125 (S)	0.3 (S)	-
19	Before treatment	0.5 (R)	0.008 (S)	0.063 (S)	0.6 (S)	-
26	Before treatment	0.5 (R)	0.063 (S)	0.25 (S)	0.6 (S)	-
43	Before treatment	0.5 (R)	0.063 (S)	0.063 (S)	0.6 (S)	DLM: Group 2_ *fbiC*_p.Ala855fs
45	During treatment	0.5 (R)	0.031 (S)	0.125 (S)	0.6 (S)	-
55	During retreatment	0.5 (R)	0.063 (S)	0.25 (S)	0.6 (S)	-
63	During treatment	0.5 (R)	0.031 (S)	0.063 (S)	0.6 (S)	-
106	Before treatment	0.5 (R)	0.063 (S)	0.031 (S)	0.6 (S)	-
127	During retreatment	0.5 (R)	0.063 (S)	0.5 (S)	0.6 (S)	-
27	Before treatment	0.25 (S)	0.25 (R)	0.25 (S)	0.6 (S)	-
28	During treatment	0.125 (S)	0.125 (R)	0.25 (S)	0.6 (S)	-
36	Before treatment	0.016 (S)	0.125 (R)	0.5 (S)	0.6 (S)	-
64	During treatment	0.25 (S)	0.125 (R)	0.5 (S)	0.6 (S)	DLM: Group 2_ *fbiC*_LoF
107	Before treatment	0.25 (S)	0.125 (R)	0.063 (S)	0.6 (S)	-
136	Before treatment	0.25 (S)	0.25 (R)	1 (S)	0.6 (S)	-
12	During treatment	0.25 (S)	0.063 (S)	>4 (R)	0.6 (S)	-
133	Before treatment	0.25 (S)	>1 (R)	>4 (R)	0.6 (S)	-
20	During treatment	0.25 (S)	0.063 (S)	0.125 (S)	1.2 (R)	DLM: Group 2_ *fbiC*_p.Ala855fs
5	Before treatment	0.016 (S)	0.063 (S)	0.5 (S)	0.6 (S)	-
6	During treatment	0.125 (S)	0.016 (S)	0.125 (S)	0.6 (S)	-
8	Before treatment	0.125(S)	0.063 (S)	0.125 (S)	0.6 (S)	-
9	During treatment	0.25(S)	0.016 (S)	0.125 (S)	0.6 (S)	-
15	Before treatment	0.25 (S)	0.008 (S)	0.031 (S)	0.3 (S)	-
21	Before retreatment	0.125 (S)	0.008 (S)	0.063 (S)	0.6 (S)	DLM: Group 2_ *fbiC*_p.Ala855fs
23	Before treatment	0.125 (S)	0.016 (S)	0.063 (S)	0.3 (S)	-
25	Before treatment	0.125 (S)	0.008 (S)	0.031 (S)	0.3 (S)	-
29	Before treatment	0.125 (S)	0.008 (S)	0.031 (S)	0.3 (S)	-
32	Before treatment	0.25 (S)	0.016 (S)	0.25 (S)	0.3 (S)	-
38	During retreatment	0.063 (S)	0.031 (S)	0.063 (S)	0.6 (S)	-
44	During treatment	0.25 (S)	0.016 (S)	0.25 (S)	0.3 (S)	-
47	Before treatment	0.016 (S)	0.063 (S)	0.25 (S)	0.6 (S)	-
51	During treatment	0.125 (S)	0.016 (S)	0.125 (S)	0.3 (S)	-
61	Before treatment	0.125 (S)	0.063 (S)	0.25 (S)	0.6 (S)	-
62	Before treatment	0.125 (S)	0.031 (S)	0.125 (S)	0.6 (S)	-
66	Before retreatment	0.25 (S)	0.031 (S)	0.125 (S)	0.3 (S)	-
67	During treatment	0.25 (S)	0.031 (S)	0.25 (S)	0.6 (S)	DLM: Group 2_ *fbiC*_p.Ala855fs
68	During retreatment	0.125 (S)	0.016 (S)	0.125 (S)	0.3 (S)	-
70	After treatment	0.25 (S)	0.016 (S)	0.063 (S)	0.3 (S)	-
75	Before treatment	0.125 (S)	0.031 (S)	0.031 (S)	0.3 (S)	-
89	Before treatment	0.125 (S)	0.016 (S)	0.25 (S)	0.6 (S)	-
92	During retreatment	0.25 (S)	0.063 (S)	0.125 (S)	0.6 (S)	-
113	Before treatment	0.25 (S)	0.063 (S)	0.5 (S)	0.6 (S)	-
134	During treatment	0.125 (S)	0.063 (S)	0.25 (S)	0.6 (S)	-
137	Before treatment	0.25 (S)	0.063 (S)	0.125 (S)	0.6 (S)	-
138	Before treatment	0.125 (S)	0.016 (S)	0.125 (S)	0.3 (S)	-
140	Before retreatment	0.25 (S)	0.031 (S)	0.125 (S)	0.6(S)	-

* Group 1, associated with resistance; ^†^ Group 2, associated with resistance—interim; R, elevated MIC; S, susceptible; BDQ, bedaquiline; DLM, delamanid; PMD, pretomanid; LZD, linezolid; gDST, genotypic drug susceptibility testing; pDST, phenotypic drug susceptibility testing.

**Table 2:** Mutations in BDQ-, DLM-, PMD-, and LZD-associated genes based om the WHO confidence-grading system.

Drugs	Gene	Amino Acid Change	Isolates (N)		2024 WHO Final Confidence Grading
			R	S	
BDQ	*mmpS5*	Val55Met	1	2	3
	*lpqB*	Asp142Gly	0	1	3
	*mmpL5*	LoF	0	2	3
		Tyr300 *	0	1	3
		Ile948Val	12	37	4
		Thr794Ile	9	33	4
		Asp767Asn	0	10	5
	*mtrB*	Met517Leu	12	37	3
		Pro18Ser	3	22	3
		c.66C > T	2	3	4
	*Rv1979c*	c.-129A > G	12	37	4
		Asp286Gly	0	1	4
DLM and PMD	*fgd1*	Arg64Ser	0	1	3
		c.774G > A	0	2	4
		c.960T > C	9	33	4
	*fbiC*	LoF	1	0	2
		Ala855fs	0	4	2
		Ala524Gly	0	3	3
		c.2427C > G	0	3	4
	*ddn*	Gly81Ser	1	0	3
	NM	-	0	2	-
LZD	*rrl*	n.1052G > T	0	1	3
		n.2160A > G	0	1	3
		n.2186G > A	0	1	3
		n.2196C > T	0	1	3
		n.2201T > C	0	1	3
		n.2496T > C	0	0	3
		n.2508T > A	0	0	3
		n.2538C > A	1	1	3
		n.2543A > T	1	1	3
		n.2547C > A	1	1	3
		n.2554G > T	1	1	3
		n.2564T > C	1	1	3
		n.2568T > G	1	1	3
		n.2595T > C	1	1	3
		n.2603A > G	1	1	3
		n.2623A > C	1	1	3
		n.2637A > G	0	1	3
		n.2640C > A	0	1	3
		n.2644A > C	0	1	3
		n.2655T > C	0	1	3
		n.2675G > C	0	1	3
		n.2680C > T	0	1	3
		n.2701T > C	0	1	3
		n.2715T > C	0	1	3
		n.2726T > A	0	1	3
	*Rv2752c*	Gly458Asp	0	1	3
	*rplC*	Phe452Leu	0	1	3
	*tsnR*	Leu232Pro	2	47	5

* stop codon (nonsense mutation); Group 1, associated with resistance; Group 2, associated with resistance—interim; Group 3, uncertain significance; Group 4, not associated with resistance—interim; Group 5, not associated with resistance; R, elevated MIC; S, susceptible; NM, no mutation; BDQ, bedaquiline; DLM, delamanid; PMD, pretomanid; LZD, linezolid.

**Table 3:** Mutations in 22 elevated-MIC isolates that are absent from the WHO catalog.

Drug	Gene	Isolates	Reference Sequence	Coding Region Change	Amino Acid Change	Location
BDQ (n = 12) *	*glpK*	3	NP_218213.1	c.1134C > T	-	Coding sequence, synonymous SNV
		1 ^†^		c.779delG	p.Gly260fs	Coding sequence, frameshift ^‡^
DLM (n = 9) and PMD (n = 2)	*fbiC*	2	NP_215689.1	c.2549C > T	p.Thr850Ile	Coding sequence, nonsynonymous SNV
		2	NP_215690.1	c.*190G > A	-	3′UTR, SNV
		2	NP_215691.1	c.*723G > A	-	3′UTR, SNV
		2	NP_215693.1	c.-3526C > T	-	5′UTR/promoter, SNV
		2	NP_215694.1	c.-3885C > T	-	5′UTR/promoter, SNV
		2	YP_177793.1	c.-2798G > A	-	5′UTR/promoter, SNV
LZD (n = 2)	Not detected	-	-	-	-	-

* The numbers in parentheses next to each drug indicate the number of isolates showing phenotypic resistance to the respective drug. ^†^ This isolate showed elevated MICs to both BDQ and DLM. ^‡^ Mutations caused by insertions or deletions altering the reading frame. 3′UTR, 3 prime untranslated region; 5′UTR, 5 prime untranslated region; SNV, single nucleotide variant; BDQ, bedaquiline; DLM, delamanid; PMD, pretomanid; LZD, linezolid.

**Table 4:** Mutations detected in 27 drug-susceptible isolates that are absent from the WHO catalog.

Drug	Gene	Isolates	Reference Sequence	Coding Region Change	Amino Acid Change	Location
BDQ	*Rv1979c*	1	NP_216495.2	c.857A > G	p.Asp286Gly	Coding sequence, nonsynonymous SNV
		1		c.768_917del	p.Pro257_Val306del *	Coding sequence deletion
		1		c.691G > A	p.Ala231Thr	Coding sequence, nonsynonymous SNV
		1		c.1016G > A	p.Arg339His	Coding sequence, nonsynonymous SNV
		1		c.1225C > T	p.Arg409 *	Coding sequence, nonsynonymous SNV
		1		c.1404C > T	-	Coding sequence, nonsynonymous SNV
	*glpK*	1	NP_218213.1	c.1379T > C	p.Val460Ala	Coding sequence, nonsynonymous SNV
		1		c.682C > G	p.Leu228Val	Coding sequence, nonsynonymous SNV
		1		c.779delG	p.Gly260fs	Coding sequence, frameshift
DLM & PMD	*fbiC*	2	NP_215689.1	c.*92_*153del	-	3′UTR, deletion
		1		c.2549C > T	p.Thr850Ile	Coding sequence, nonsynonymous SNV
		1		c.1571C > G	p.Ala524Gly	Nonsynonymous SNV
		1		c.2162C > T	p.Ala721Val	Nonsynonymous SNV
		1		c.2427C > G	p.Tyr809 *	Nonsense SNV
LZD	*rrl*	1	NP_215830.1	c.-4457C > A	-	Non-coding SNV, intergenic or upstream variant
		1	NP_215832.1	c.*1814A > G	-	Non-coding SNV, downstream variant
	*rplD*	1	NP_215216.1	c.189G > A	p.Arg79His	Coding sequence, nonsynonymous SNV
	*rplV*	1	NP_215220.1	c.42C > T	-	Coding sequence, nonsynonymous SNV

* Deletion of nucleotide(s) at specified position(s); 3′UTR, 3 prime untranslated region; SNV, single nucleotide variant; BDQ, bedaquiline; DLM, delamanid; PMD, pretomanid; LZD, linezolid.

## Data Availability

The whole-genome sequencing data generated in this study have been deposited in the NCBI Sequence Read Archive (SRA) under BioProject accession number PRJNA1301050.

## Abbreviations

The following abbreviations are used in this manuscript:

BDQ	bedaquiline
DLM	delamanid
gDST	phenotypic drug susceptibility testing
INH	isoniazid
LVX	levofloxacin
LZD	linezolid
MDR-TB	multidrug-resistant tuberculosis
MFX	moxifloxacin
MIC	minimum inhibitory concentration
pDST	phenotypic drug susceptibility testing
PMD	pretomanid
REMA	resazurin microdilution assay
RIF	rifampicin
RR-TB	rifampicin-resistant TB
TB	tuberculosis
WGS	whole-genome sequencing
WHO	World Health Organization
XDR-TB	extensively drug-resistant tuberculosis
